# MicroRNA-Mediated Down-Regulation of Apoptosis Signal-Regulating Kinase 1 (ASK1) Attenuates the Apoptosis of Human Mesenchymal Stem Cells (MSCs) Transplanted into Infarcted Heart

**DOI:** 10.3390/ijms17101752

**Published:** 2016-10-20

**Authors:** Chang Youn Lee, Sunhye Shin, Jiyun Lee, Hyang-Hee Seo, Kyu Hee Lim, Hyemin Kim, Jung-Won Choi, Sang Woo Kim, Seahyung Lee, Soyeon Lim, Ki-Chul Hwang

**Affiliations:** 1 Department of Integrated Omics for Biomedical Sciences, Yonsei University, 03722 Seoul, Korea; cylee083@gmail.com (C.Y.L.); ssh5043@naver.com (S.S.); kimhyaemin17@gmail.com (H.K.); 2 Brain Korea 21 PLUS Project for Medical Science, Yonsei University, 03722 Seoul, Korea; jylee12@yuhs.ac (J.L.); shh17@yuhs.ac (H.-H.S.); 3 Department of Veterinary Medicine, Chonbuk National University, 54896 Jeonju, Korea; gurrygurry@naver.com; 4 Institute for Bio-Medical Convergence, College of Medicine, Catholic Kwandong University, Gangneung, 25601 Gangwon-do, Korea; gardenia@hanmail.net (J.-W.C.); doctor7408@gmail.com (S.W.K.); sam1017@ish.ac.kr (S.L.); 5 Catholic Kwandong University, International St. Mary’s Hospital, 22711 Incheon, Korea

**Keywords:** apoptosis signal-regulating kinase 1 (ASK1), mesenchymal stem cells (MSCs), adipose-derived stem cell (ASC), reactive oxygen species (ROS), myocardial infarction (MI), microRNA-301a

## Abstract

Stem cell therapy using adult stem cells, such as mesenchymal stem cells (MSCs) has produced some promising results in treating the damaged heart. However, the low survival rate of MSCs after transplantation is still one of the crucial factors that limit the therapeutic effect of stem cells. In the damaged heart, oxidative stress due to reactive oxygen species (ROS) production can cause the death of transplanted MSCs. Apoptosis signal-regulating kinase 1 (ASK1) has been implicated in the development of oxidative stress-related pathologic conditions. Thus, we hypothesized that down-regulation of ASK1 in human MSCs (hMSCs) might attenuate the post-transplantation death of MSCs. To test this hypothesis, we screened microRNAs (miRNAs) based on a miRNA-target prediction database and empirical data and investigated the anti-apoptotic effect of selected miRNAs on human adipose-derived stem cells (hASCs) and on rat myocardial infarction (MI) models. Our data indicated that miRNA-301a most significantly suppressed ASK1 expression in hASCs. Apoptosis-related genes were significantly down-regulated in miRNA-301a-enriched hASCs exposed to hypoxic conditions. Taken together, these data show that miRNA-mediated down-regulation of ASK1 protects MSCs during post-transplantation, leading to an increase in the efficacy of MSC-based cell therapy.

## 1. Introduction

Ischemic heart diseases are one of the leading causes of morbidity and mortality worldwide. Myocardial infarction (MI) leads to a loss of cardiomyocytes, resulting in left ventricular remodeling and decreased cardiac function [1]. Although traditional treatments for MI, such as physical surgery and medicinal treatment, have contributed to improvements, they remain insufficient for regenerating areas of ischemic damage in the heart. These treatments are only capable of delaying the progression of heart failure because the damaged myocardial cells cannot be fundamentally repaired [2]. For this reason, various treatments have been suggested for MI therapy, such as genes, cytokines, or cell-based treatments, as well as modified traditional treatments [3].

In the last decade, various types of stem cells have been proposed as a promising therapy for the regeneration of damaged tissues; among them, the therapeutic effects of mesenchymal stem cells (MSCs) in treating various diseases have been extensively reported. MSCs, which are multipotent cells, can differentiate into various cell types and can be easily isolated from various adult and fetal tissues, such as adipose, bone marrow, synovial fluid, umbilical cord blood, and amniotic fluid [4,5,6]. Nevertheless, the low survival of MSCs transplanted into damaged tissue under harsh conditions remains an unsolved issue, and MSCs have not been capable of presenting distinct therapeutic effects [7,8]. Adipose-derived stem cells (ASCs) have been reported to be easily isolated from adipose tissue and to have properties of MSCs, including self-renewal and pluripotent properties [9]. Due to these useful characteristics of ASCs, interest in applications using ASCs has increased. Therefore, we expect that ASCs may be an ideal source of stem cells for cardiac therapy [10].

Apoptosis signal-regulating kinase 1 (ASK1), a serine/threonine protein kinase, is a member of the MAPKKK family and is known to be activated by a variety of stresses, such as reactive oxygen species (ROS) and cell death ligands (Fas and TNF) [11,12,13]. ASK1 has been shown to be associated with apoptosis in several tissues, such as cancer and heart tissues. ASK1 was previously shown to have an important role as an upstream regulator of p38 and JNK in cardiomyocytes death [14]. Down-regulation of ASK1 expression has an anti-apoptotic effect on cancer cells [11,15]. Although studies in the last two decades revealed that ASK1 is an important regulator of apoptosis, the association between ASK1 and stem cells has rarely been investigated despite the expected effect of ASK1 on stem cell survival.

MicroRNAs (miRNAs) are short, non-coding, and single-stranded RNA sequences that consist of approximately 18–22 nucleotides and that regulate their target genes by binding the complementary 3′ untranslated region (3′ UTR) of mRNAs, resulting in translational repression or degradation [16]. During the last decade, several studies have revealed that miRNAs control various biological functions, including development, differentiation, proliferation, and apoptosis [17,18,19].

In this study, we investigated the overexpression of miRNA-301a in human ASCs (hASCs) and found that this miRNA increased cell survival by inhibiting ASK1 expression under hypoxic conditions. We also observed that the expression level of ASK1 was associated with cell death, and that miRNA-301a overexpression could attenuate the activation of hypoxia-induced apoptotic signaling in hASCs. These results suggest that miRNA-mediated fortification of hASCs, which results in increased cell survival, is a novel potential therapeutic target or approach to developing strategies for treating an infarcted heart.

## 2. Results

### 2.1. Hypoxia Induced Apoptosis Signal-Regulating Kinase 1 (ASK1) Activation in Human Adipose-Derived Stem Cells (hASCs)

**Figure 1:**
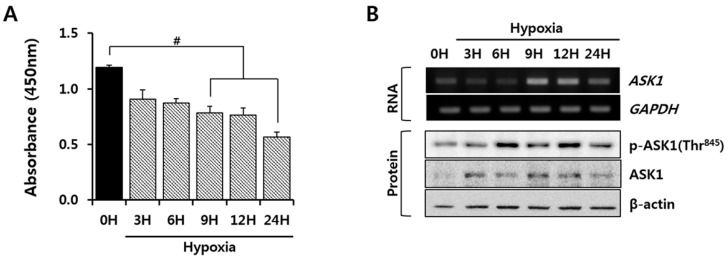
Increased expression of apoptosis signal-regulating kinase 1 (ASK1) in human adipose-derived stem cells (hASCs) exposed to hypoxic conditions. hASCs were exposed to hypoxic conditions (1% ≥ O_2_, 5% CO_2_, 37 °C) for up to 24 h. (**A**) Viability of hypoxic hASCs was measured by cell counting kit-8 reagent (CCK-8) assay (^#^ *p* < 0.05 vs. 0 h); (**B**) ASK1 mRNA and protein expression were measured by reverse transcription polymerase chain (RT-PCR) and immunoblot, respectively.

First, we investigated the viability of hASCs under hypoxic conditions that mimic in vivo transplantation into infarcted hearts. As shown in Figure 1A, cell viability was assessed for 24 h, and an up to 50% decrease in cell viability was observed under hypoxic conditions. As ASK1 is known to be an important signaling molecule that induces cell death in other cell types under oxidative stress conditions [20], we examined ASK1 mRNA and protein expression to identify whether the ASK1 expression is regulated by hypoxia. Hypoxia up-regulated ASK1 mRNA and protein expression levels in a time-dependent manner (Figure 1B). Next, we investigated whether increased ASK1 expression itself can cause cell death and confirmed that significant cell death was induced by ASK1 overexpression (Figure S1). Moreover, we demonstrated ASK1 phosphorylation of Thr845, which has been reported to be essential for ASK1 activation and apoptosis due to stress, such as oxidative stress [21]. Hypoxia significantly increased the phosphorylation of ASK1 at Thr845 (Figure 1B). These results suggest an association between hypoxia-mediated cell death and the expression and activation of ASK1 in hASCs.

### 2.2. miRNA-301a Targeted ASK1 in hASCs

**Figure 2:**
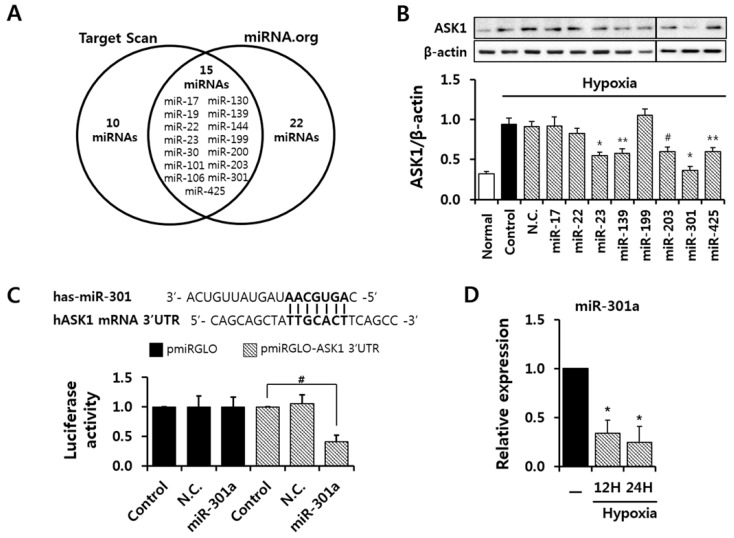
Screening of predicted miRNAs for targeting ASK1. (**A**) Candidate miRNAs that target ASK1 were selected based on prediction databases (www.TargetScan.org & www.microRNA.org); (**B**) ASK1 expression in ASCs transfected with the indicated candidate miRNAs was analyzed by immunoblot (^#^ *p* < 0.05, ** *p* < 0.01, * *p* < 0.001 vs. Control, N.C.: negative control); (**C**) Luciferase assay using the 3’untranslated region (3′ UTR) of ASK1 was performed to confirm the interaction between miRNA-301a and ASK1 (^#^ *p* < 0.05 vs. Control); (**D**) Endogenous expression of miRNA-301a was measured by real-time PCR (* *p* < 0.001 vs. Control).

To evaluate the anti-apoptotic effect of ASK1 inhibition, we screened 15 candidate miRNAs that target ASK1 as predicted by miRNA-target prediction databases (http://www.targetscan.org, http://www.microrna.org) and shown in Figure 2A. We investigated the protective effects of ASK1 inhibition by candidate miRNA transfection on cell death under hypoxic conditions, and we found that around eight miRNAs, including miR-301a overexpression, showed the protective effect against cell death (Figure S2). Next, then we transfected these miRNAs to check whether they can regulate ASK1 protein expression levels in hASCs (Figure 2B). As the basal protein level of ASK1 is low, ASCs were subjected to hypoxic conditions after miRNA transfection. Several miRNAs, including miRNA-17, -139, and -301a, were capable of down-regulating the ASK1 protein level. We found that the miRNA-301a binding site is highly conserved in the 3′ UTR of ASK1 mRNA. Luciferase assay using vectors containing the 3′ UTR of ASK1 confirmed that miRNA-301a targets ASK1 (Figure 2C). In addition, we confirmed that endogenous miR-301a expression decreased until 24 h in hASCs under hypoxic conditions (Figure 2D).

### 2.3. miRNA-301a Has Anti-Apoptotic Effects on hASCs under Hypoxic Conditions

**Figure 3:**
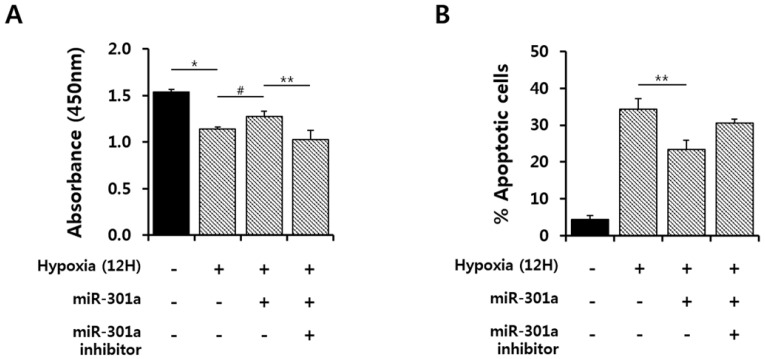
miRNA-301a attenuated cell death under hypoxic conditions. miRNA-301a was transfected into hASCs under hypoxic conditions for 12 h. (**A**) The cell viability rate was measured by cell counting kit-8 reagent (CCK-8) assay (^#^ *p* < 0.05 vs. hypoxia, ** *p* < 0.01 vs. inhibitor, * *p* < 0.01 vs. Normal); (**B**) The number of apoptotic cells decreased as detected by annexin V/PI after miR-301 transfection in vitro (annexin V/PI). The values are the average of three measurements, and the S.E. is indicated by error bars (** *p* < 0.05 vs. hypoxia).

To determine whether miRNA-301a regulates cell survival by targeting ASK1 under hypoxic conditions, cell viability was examined under hypoxic conditions, followed by treatment with a miR-301a mimic or inhibitor. miRNA-301a mimic treatment showed a significant protective effect against cell death, but a miRNA-301a inhibitor blocked the protective effect of miRNA-301a overexpression (Figure 3A). In addition, we further investigated the protective effect using siRNA for ASK1 under hypoxic conditions, as ASK1 is one of the major targets of miRNA-301a (Figure S3). Cell death was significantly attenuated by ASK1 siRNA treatment under hypoxic conditions. This result demonstrates that ASK1 is one of important factor to regulate cell survival. Then, we used an annexin V assay to test the anti-apoptotic effect of miRNA-301a under hypoxic conditions and confirmed the anti-apoptotic effect of ASK1 inhibition using miRNA-301a under hypoxic conditions (Figure 3B and Figure S4).

### 2.4. miRNA-301a Represses the Apoptotic Pathway via Down-Regulation of the ASK1-Mediated Signaling Pathway during Hypoxia

**Figure 4:**
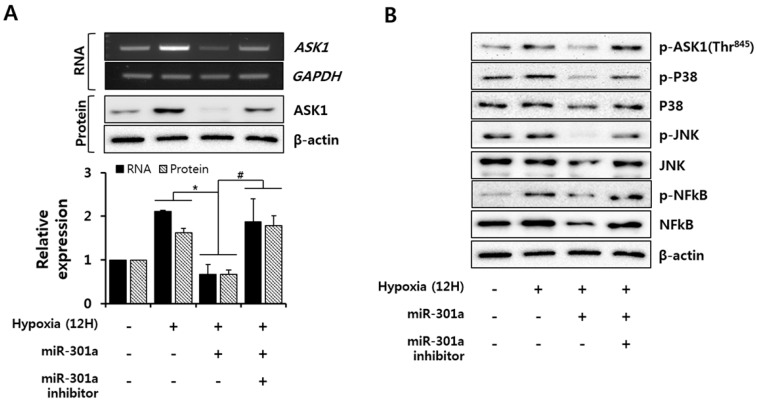
miRNA-301a inhibits the ASK1-related apoptotic pathway. (**A**) ASK1 mRNA and protein expression were measured by RT-PCR and immunoblot (* *p* < 0.001 vs. hypoxia, ^#^ *p* < 0.05 vs. inhibitor); (**B**) Expression and phosphorylation levels of ASK1-downstream molecules (JNK, p38, and NFκB) were detected by immunoblot.

To investigate the signaling molecules that are regulated by miRNA-301a, which target ASK1 under hypoxic conditions, we first examined the ASK1 mRNA and protein expression levels under hypoxic conditions with or without miRNA-301a overexpression. Hypoxia consistently caused increased ASK1 mRNA and protein expression levels, as shown in Figure 1, whereas miRNA-301a mimic treatment caused decreases in ASK protein expression and phosphorylation as well as ASK1 mRNA expression. Additionally, these effects were reversed by miR-301a inhibitor treatment of these cells (Figure 4A,B). ASK1 acts as an upstream regulator of p38 and JNK activation [22]. To determine whether miRNA-301a regulates the ASK1-mediated apoptotic pathway, we examined JNK and p38 activation under hypoxic conditions with or without miRNA-301a overexpression. Hypoxia led to p38 and JNK phosphorylation, which was inhibited by miRNA-301a overexpression, and miRNA-301a inhibitor treatment interrupted the inhibitory effect of miRNA-301a on p38 and JNK phosphorylation (Figure 4B). As an apoptosis-associated transcription factor, NFκB was investigated to determine the anti-apoptotic effect of ASK1 inhibition by miRNA-301a overexpression. Hypoxic stress resulted in NFκB phosphorylation, whereas miRNA-301a mimic treatment attenuated NFκB phosphorylation; this effect was reversed by miRNA-301a inhibitor treatment (Figure 4B). These inhibition effects for signaling molecules due to ASK1 inhibition were also confirmed by siRNA treatment (Figure S5). In addition to the localization of ASK1 in cytoplasm, ASK1 is known to be localized in mitochondria and to be able to induce mitochondrial-dependent apoptosis [23]. Therefore, we further examined caspase 3 activation to determine whether miRNA-301a inhibits the mitochondrial-dependent apoptosis mediated by ASK1. miRNA-301a was not able to inhibit caspase 3 activation (Figure S6). Our data indicated that miRNA-301a suppressed the hypoxia-induced expression and activation of proapoptosis-related factors (JNK, p38, and NFκB).

### 2.5. Effect of hASC^miR-301^ on Ischemic Myocardium

**Figure 5:**
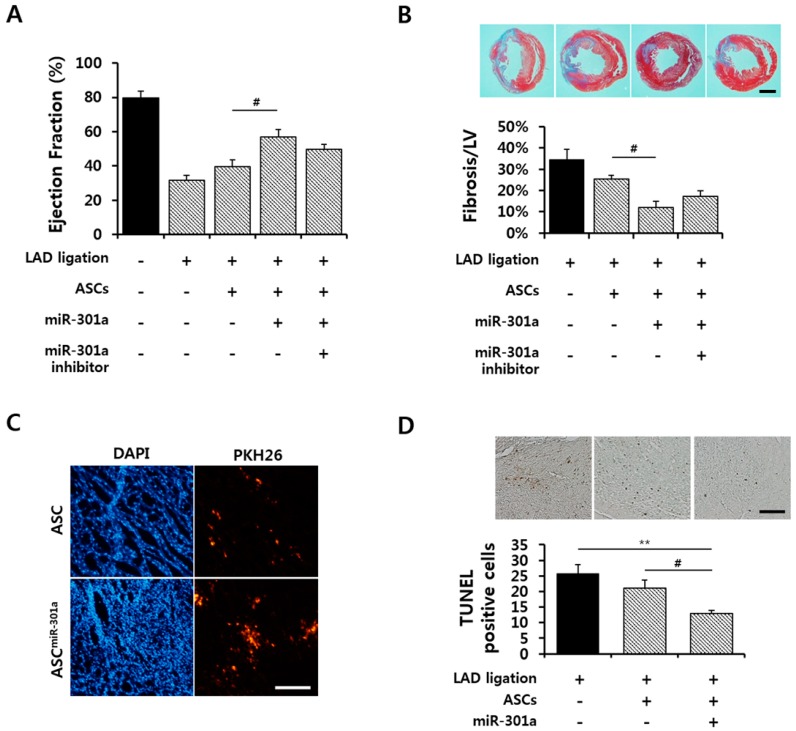
Effects of hASC^miR-301^ on cardiac function after myocardial infarction (MI). (**A**) Cardiac functions were assessed using a Millar micro-tip 2 F pressure transducer at one week after MI (^#^ *p* < 0.05); (**B**) Fibrosis was detected by Masson’s trichrome staining from three rats per group. Scale bar = 5 mm (^#^ *p* < 0.05); (**C**) One week after the ASCs injection, PKH26-stained ASCs were detected in the ischemic myocardium. Scale bar = 200 μm; (**D**) Representative histological sections of ischemic myocardium stained with TUNEL assay at one week after MI. Quantitative analysis was performed for TUNEL (terminal deoxynucleotidyltransferase-mediated dUTP nick-end labeling) positive cells. Scale bar = 100 μm (** *p* < 0.01, ^#^ *p* < 0.05).

To determine whether miRNA-301a-transfected hASCs (hASCs^miR-301^) have a therapeutic effect on ischemic myocardium, cardiac functional improvements by hASCs^miR-301^ were analyzed in normal and MI rat hearts after hASC^miR-301^ transplantation. One week after transplantation, hASCs^miR-301^ showed a significantly increased ejection fraction compared to MI and hASCs transplanted MI hearts (Figure 5A). Trichrome staining showed results that were consistent with the cardiac function data. The fibrosis area was significantly reduced by hASCs^miR-301^ injection in ischemic hearts (Figure 5B). Figure 5C shows that transplanted hASCs^miR-301^ had greater survivorship rates than transplanted hASCs at injected sites, with less cell death observed in hASCs^miR-301^ injected heart tissue (Figure 5D).

## 3. Discussion

We used a miRNA that targets ASK1 as a regulatory tool to modulate ASK1 expression and stem cell activation under hypoxic conditions and investigated the regulatory effect of miRNA-301a on ASK1-mediated apoptosis in hASCs. In this study, exposing hASCs to hypoxic conditions led to ASK1 activation, resulting in the apoptosis of these cells. Additionally, miRNA-301a treatment was capable of attenuating hASCs apoptosis by regulating cytosolic in hypoxia-treated cells and in a MI heart.

We observed that ASK1 is activated under hypoxic conditions (Figure 1). ASK1 is known to be activated by ER stress, calcium influx, and oxidative stress, which all are related to hypoxic conditions [20]. Various regulatory proteins are known to co-regulate ASK1 phosphorylation under ROS stress. TNF-α receptor-associated factor 2 (TRAF2) and TRAF6 positively regulate ASK1 activity, promoting cell death, whereas protein phosphatase 5 can negatively regulate ASK1 activity, resulting in ASK1 dephosphorylation [24]. Although we did not investigate these proteins which can support ASK1 function in the present study, miRNA-301a inhibited ASK1 phosphorylation through transcriptional and translational repression of ASK1, resulting in the attenuation of cell death (Figure 3 and Figure 4). Here, we investigated p38/JNK/NFκB signaling molecules as important downstream signaling pathways for ASK1. Although p38, JNK, and NFκB are not specific signaling molecules for ASK1, these have been known as major signaling molecules activated by ASK1 under ROS stress [11]. In this study, we showed ASK1-dependent downregulation of p38/JNK/NFκB activation using ASK1 siRNA (Figure S5). Moreover, studies using inhibitor compounds targeting ASK1-p38-JNK pathways showed potential therapeutic effects for the ASK1-related diseases including vascular diseases, neurodegenerative disorders, and inflammatory diseases [20,25,26,27]. As another downstream signaling molecule, NFκB has been known to act differently in different stimulus [28]. Under hypoxia, NFκB induced apoptosis by suppressing bcl-2 and NFκB inhibitor treatment was capable to prevent apoptosis [29]. Another study also suggested that NFκB activation is important for oxidative stress-induced apoptosis under hypoxia and showed that suppression of NFκB activation attenuated apoptosis through attenuation of p53 expression [30].

In this study, we primarily investigated the role of ASK1 in transplanted stem cells and their survival mechanism by miRNA-301a under hypoxic conditions which produces ROS and subsequent apoptosis. Moreover, the regulation of ASK1 expression and activation by miRNA-301a considerably improved stem cell survival and increased ischemic heart function (Figure 5). However, the heart consists of several other major cell types, such as cardiomyocytes, cardiac fibroblasts, endothelial cells, and smooth muscle cells; these cells can be affected by hypoxic conditions and then create a harsh environment for stem cell survival after transplantation. Indeed, several studies have reported that transfecting miRNAs or endogenous miRNAs of stem cells can be released or transferred into neighboring cells by exosomes and suggested that this may be the future of biological therapy. Monoz et al. demonstrated that miRNA-9 could be transferred from MSCs to cancer cells through MSC-derived exosomes [31]. Another study showed that the administration of exosomes containing miRNA-146 from MSCs reduced brain tumors [32]. In our study, as miRNA-301a expressed by stem cells could be released into the adjacent environment, which includes other cell types, determining the effect of miRNA-301a on regulating ASK1 expression in other cardiac cell types is important. Although few studies have reported on an association between miRNA-301a and ASK1 in cardiomyocytes, the role of ASK1 in apoptosis has been well established. miRNA-320, which targets ASK1, suppressed cardiomyocyte apoptosis by down-regulating ASK1/JNK phosphorylation under ischemia/reperfusion injury [33]. In endothelial cells, ASK1 inhibition significantly attenuates JNK-dependent and JNK-independent apoptosis. ASK1 is regulated by binding to cytosolic thioredoxin-1 and mitochondrial thioredoxin-2 under normal conditions, and these proteins dissociate under ROS induction [23]. Another miRNA that targets ASK1, miRNA-19a, also showed an anti-apoptotic effect under lipopolysaccharide stimulation in endothelial cells [34]. miRNA-301a was also reported to be related to vascular dysfunction in human pulmonary endothelial cells [35]. Cardiac fibroblasts are known to be activated by several stimuli and activated and transformed myofibroblasts are known to lead to cardiac fibrosis, resulting in cardiac dysfunction [36]. In human fibrotic disease, miRNA-215 suppresses fibroblast proliferation by targeting ASK1; several miRNAs targeting ASK1 were introduced as new therapeutic targets for kidney fibrosis [37,38]. Considering these findings, we speculate that controversial effects may not occur among different cell types, and we anticipate a consistent effect of miRNA-301a regulation of ASK1 expression and activation on hASCs^miR-301^ transplanted into infarcted hearts.

## 4. Materials and Methods

### 4.1. Culture of hASCs

Human adipose derived stem cells (hASCs) were purchased from Invitrogen (Waltham, MA, USA). hASCs were cultured according to the manufacturer’s instructions. We used high glucose-Dulbecco’s modified Eagle’s medium (DMEM; Gibco, Waltham, MA, USA) containing 10% fetal bovine serum (FBS; Gibco) and 1% antibiotics (Gibco). The media were changed every three days, and cells were passaged using 0.25% trypsin (Gibco) when they reached 80% to 90% confluency. Cells from passages 6 to 10 were used for experiments.

### 4.2. Induction of Hypoxia in hASCs

To induce hypoxia, 80% confluent hASCs were plated in cell culture dishes. The serum-free media were degassed and then exposed in a hypoxic chamber (Thermo Fisher Scientific, Waltham, MA, USA) maintained below 1% O_2_ concentration at 37 °C.

### 4.3. Transfection

(i) miRNA: Transfection with miRNA-301a mimics or miRNA-301a inhibitors was performed using the TransIT-X2 system (Mirus Bio, Madison, WI, USA). Mature miRNA-301a mimics (Genolution Pharmaceuticals, Seoul, Korea) and miRNA-301a inhibitors (Integrated DNA Technologies, Coralville, IA, USA) were used at final concentrations of 50 nM. After the cells were incubated for 24 h, the media were replaced with fresh ones for stabilization.

(ii) siRNA: Transfection of siRNA was performed using the TransIT-X2 system. Commercial AccuTarget siRNAs, (Bioneer, Daejeon, Korea) which are human ASK1 siRNA (sense 5′-GACAUCAGGAAAGCUCGUA (dTdT)-3′; antisense 5′-UACGAGCUUUCCUGAUGUC (dTdT)-3′), were designed to knockdown human ASK1 gene expression. siRNA was used at final concentrations of 100 nM.

(iii) Plasmid: Human ASK1 expression plasmids were obtained from Addgene (Cat.No.# 47104). The plasmids were delivered to the cells using TransIT-2020 system (Mirus Bio), with 1 µg of plasmid used for each 60 mm culture plate.

### 4.4. Cell Viability Assay

To measure cell viability, 5 × 10^3^ hASCs cells were plated in a 96-well plate. The cells were transfected with 50 nM miRNA mimics and incubated for 24 h. The plate was exposed to hypoxic conditions for 12 h. Then, cell counting kit-8 reagent (CCK-8, Dogen, Seoul, Korea) was added to each well to a final concentration of 0.5 mg/mL, and the cells were incubated for two hours. The absorbance at 450 nm was measured using a microplate reader (Thermo Fisher Scientific).

### 4.5. Reverse Transcription Polymerase Chain Reaction (RT-PCR)

In total, 2 × 10^5^ hASCs were plated in six-well plates and exposed to hypoxic conditions for six hours. Total RNA was extracted using TRIzol reagent (Ambion, Waltham, MA, USA) according to the manufacturer’s instructions. Complementary DNA (cDNA) was synthesized from RNA by AMV reverse transcriptase in provided in a RT system kit (Promega, Fitchburg, WI, USA). PCRs were performed for 35 cycles with primers based on the ASK1 gene sequence. The primer sequences were as follows: ASK1, sense: 5′-CGTAGCCTCTTGGTCCTTTATC-3’, 5′-GGAAGTCTTTCTGCTCTCCTTC-3′; GAPDH, sense: 5′-CATGGGTGTGAACCATGAGAA-3′, 5′-GGTCATGAGTCCTTCCACG AT-3′. PCR conditions were set to 95 °C for 3 min; followed by 35 cycles of 95 °C for 30 s, 62 °C for 30 s, 72 °C for 90 s; and a final extension at 72 °C for 10 min. Products were separated by electrophoresis in 1.2% agarose gels, and visualized using a Gel-Doc (Bio-Rad, Hercules, CA, USA) following staining with ethidium bromide.

### 4.6. Western Blot Analysis

In total, 3 × 10^5^ hASCs were plated in 60 mm dishes and exposed to hypoxic conditions for six hours. The cells were washed in PBS and then lysed in lysis buffer (Cell signaling Technology, Danvers, MA, USA), 1% phosphatase inhibitors, and 1% protease inhibitors. Protein concentrations were determined using the BCA Protein Assay kit (Thermo Fisher Scientific). Briefly, 35 µg of protein was subjected to 8% or 10% sodium dodecyl sulfate-polyacrylamide gel electrophoresis (SDS-PAGE) and then transferred to a polyvinylidene difluoride membrane (Millipore, Billerica, MA, USA). The membrane was blocked with Tris-buffered saline/Tween 20 (TBS-T, 0.05% Tween 20) and 5% skim milk for one hour at room temperature and then incubated with the appropriate primary antibodies overnight at 4 °C. The following antibodies were used in these experiments: anti-ASK1 (#8662, Cell Signaling), anti-phospho-ASK1 (#3765, Cell Signaling), anti-p38 (#9212, Cell signaling), anti-phospho-p38 (#9211, Cell Signaling), anti-JNK (#9252S, Cell Signaling), anti-phospho-JNK (#9251, Cell Signaling), anti-NFκB p65 (#8242S, Cell Signaling), anti-phospho-NFκB p65 (#3031, Cell signaling), anti-caspase3 (AB3623, Millipore), anti-β-actin (A5316, Sigma Aldrich, St. Louis, MO, USA). The membrane was washed three times with 0.01% TBS-T for five minutes and then incubated with skim milk and horseradish peroxidase-conjugated secondary antibodies (Santa Cruz Biotechnology, Dallas, TX, USA) for one hour at RT. After the membrane was washed six times for five minutes each, the bands were detected with an enhanced chemiluminescence (ECL) reagent (Santa Cruz Biotechnology). The band intensities were quantified using a Davinch-Western imagine system (Davinch K, Seoul, Korea) and NIH ImageJ version 1.44p software (National Institutes of Health, New York, NY, USA).

### 4.7. Real-Time Quantitative Polymerase Chain Reaction (qRT-PCR)

In brief, 100 ng purified total RNA was used for reverse transcription (TaqMan^®^ MicroRNA Reverse Transcriptase Kit, Applied Biosystems, Waltham, MA, USA) in combination with TaqMan MicroRNA Assays to quantify miRNA-301a and U6 control transcripts according to the manufacturer’s conditions. The threshold cycle (*C*_t_) of miR-301a and U6 expression was automatically defined, located in the linear amplification phase of the PCR, and normalized to the control U6 (Δ*C*_t_ value). The relative difference in the expression level of miR-301a in the sorted cells (ΔΔ*C*_t_) was calculated and presented as the fold induction (2^–ΔΔ*C*t^).

### 4.8. Annexin V/PI Apoptosis Assay

To quantify apoptosis, 3 × 10^5^ hASCs were plated in 60 mm dishes and exposed to hypoxic conditions for 12 h. Then the cells were washed twice with PBS, and 1 × 10^5^ cells were resuspended in 100 µL of 1× binding buffer containing annexin V-FITC and propidium iodide (Apoptosis Detection kit, BD Biosciences, Franklin Lakes, NJ, USA) and then incubated for 15 minutes in the dark at RT. Next, 2 × 10^4^ cells were analyzed by flow cytometry (BD ACCURI C6 cytometer, BD Biosciences). Annexin V−/PI− staining indicated the viable cells, annexin V+/PI− indicated the early apoptotic cells, and annexin V+/PI+ staining indicated the necrotic or late apoptotic cells.

### 4.9. Luciferase Assay

The 3′ UTR sequence of ASK1 was amplified using primers with XhoI (forward) and Xba1 (reverse) endonuclease sites. The 3′ UTR fragment was then cloned into the pMIR GLO vector. HeLa cells were plated at a density of 1 × 10^5^ cells/well in a 12-well plate and then transfected with either pMIR GLO control vector or pMIR GLO vector with ASK1 3′ UTR using Lipofectamine 2000. Luciferase activity was measured 48 h later using a luminometer and Dual Luciferase Assay Kit (Promega Corporation, Fitchburg, WI, USA) according to the manufacturer’s instructions. Renilla luciferase (Promega Corporation) was used to normalize the cell number and transfection efficiency.

### 4.10. Induction of Myocardial Infarction and Cell Transplantation

All experimental procedures for animal studies were approved by the Committee for the Care and Use of Laboratory Animals of Catholic Kwandong University College of Medicine (CKU01-2015-003-1) and performed in accordance with the Committee’s Guidelines and Regulations for Animal Care. Seven-week-old male Sprague-Dawley rats (220 ± 30 g) were used for in vivo experiments. After anesthetization via intraperitoneal injection of zoletil (30 mg/kg) and xylazine (10 mg/kg), rats were ventilated via trachea using a ventilator (Harvard Apparatus, Holliston, MA, USA) and then were subjected to surgically induced MI, followed by cell transplantation. The rats were randomized into five groups (normal, MI Control, ASC, ASC + 301a, and ASC + 301a inhibitor). MI was produced by surgical occlusion of the left anterior descending coronary artery by ligation using a 7-0 Prolene suture (Covidien, Dublin, Ireland). For cell transplantation, PKH26-labelled cells were suspended in 30 µL of PBS (1 × 10^6^ cells) and transplanted into the viable myocardium bordering the infarction at three injection sites using an insulin syringe (BD Ultra-Fine II, 0.3 mL) with a 30-gauge needle. To determine whether cell transplantation could have a therapeutic effect after MI, we performed trichrome staining at one week after MI and cell transplantation.

### 4.11. Terminal Deoxynucleotidyltransferase-Mediated dUTP Nick-End Labeling Assay

A terminal deoxynucleotidyltransferase-mediated dUTP nick-end labeling (TUNEL) assay was performed according to the manufacturer’s instructions (Cat.No.#S7100, Millipore). Heart tissues were fixed in 4% formaldehyde and embedded in paraffin. Tissue sections (5 μm thickness) were deparaffinized, dehydrated, and rinsed with PBS. The slides were treated with 3% hydrogen peroxide and TdT enzyme at room temperature for one hour followed by digoxygenin-conjugated nucleotide substrate at 37 °C for 30 min. Nuclei were stained with DAB (Vector Laboratories, Burlingame, CA, USA) for five minutes, and the slides were counterstained with 0.5% methyl green solution (Sigma Aldrich, St. Louis, MO, USA). The slides were observed by a virtual microscopy (BX51/dot Slide; Olympus, Tokyo, Japan).

### 4.12. Statistical Analysis

The data are expressed as the mean ± standard error of the mean of at least three independent experiments. Comparisons between more than two groups were performed by one-way analysis of variance using Bonferroni’s correction. *p* < 0.05 was considered significant.

## 5. Conclusions

This study is the first demonstration of ASK1 modulation using a miRNA in transplanted stem cells in a MI model and suggests that the suppression of ASK1 by miRNA-301a is a promising approach to prevent massive death of stem cells after transplantation.

## Supplementary Materials

Supplementary materials can be found at www.mdpi.com/1422-0067/17/10/1752/s1.
