# Supplementary material for: MicroRNA-Mediated Down-Regulation of Apoptosis Signal-Regulating Kinase 1 (ASK1) Attenuates the Apoptosis of Human Mesenchymal Stem Cells (MSCs) Transplanted into Infarcted Heart
Source: Int J Mol Sci. 2016 Oct 20;17(10):1752. doi: 10.3390/ijms17101752 (PMC5085777; doi:10.3390/ijms17101752)
Supplement: Supplementary file 1 [file ijms-17-01752-s001.pdf]

Supplementary Materials: MicroRNA-Mediated Down-Regulation of ASK1 Attenuates the Apoptosis of Human MSCs Transplanted into Infarcted Heart

Chang Youn Lee, Sunhye Shin, Jiyun Lee, Hyang-Hee Seo, Kyu Hee Lim, Hyemin Kim, Jung-Won Choi, Sang Woo Kim, Seahyung Lee, Soyeon Lim and Ki-Chul Hwang

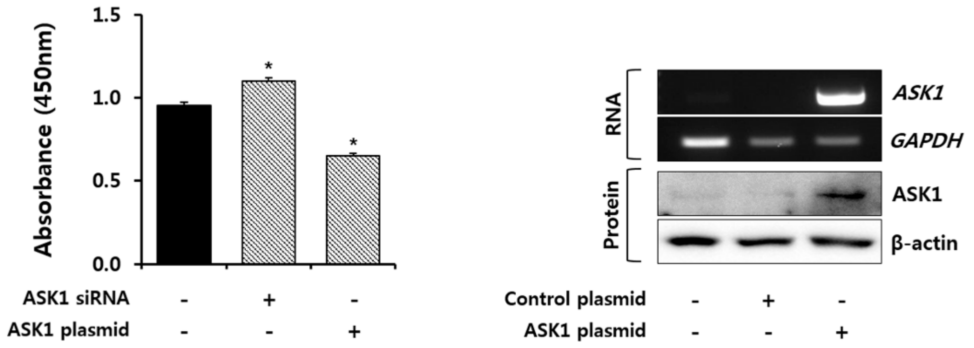

Figure S1. (**Left**) The cell viability was measured by CCK-8 assay after transfection using ASK1 siRNA or ASK1 plasmid for 24 h. (* $p < 0.001$ vs. Control); (**Right**) ASK1 mRNA and protein expressions were measured by RT-PCR and immunoblot after ASK1 plasmid transfection.

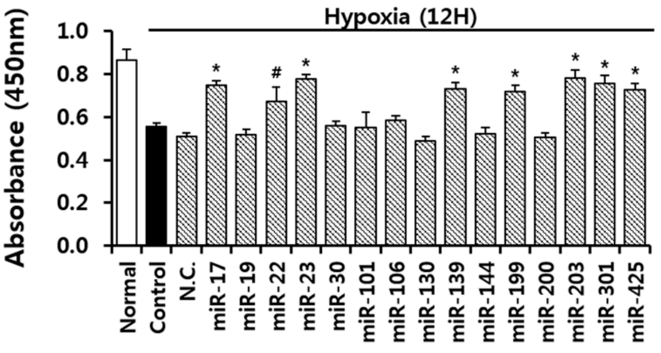

Figure S2. Effect of selected overexpressed miRNAs on hASCs survival under hypoxic conditions was evaluated by the CCK-8 assay. All samples were exposed to hypoxic conditions at 12 h after miRNA transfection. ($^{\#}$ $p < 0.05$, * $p < 0.001$ vs. Control).

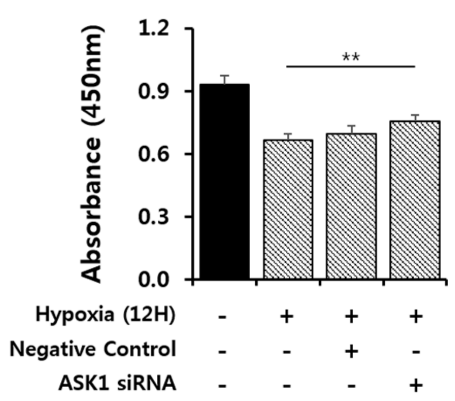

Figure S3. The cell viability was measured by CCK-8 assay followed by ASK1 siRNA transfection. (** $p < 0.01$).

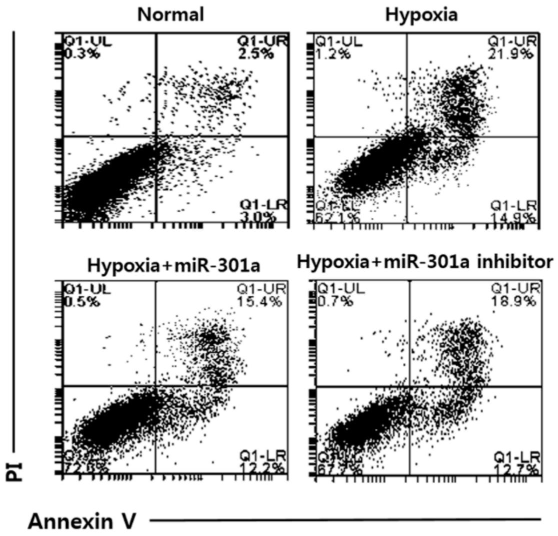

Figure S4. Annexin V/PI assay was applied to hASCs in various conditions: normal, hypoxia, hypoxia with miR-301a mimic, and hypoxia with miR-301a mimic and inhibitor.

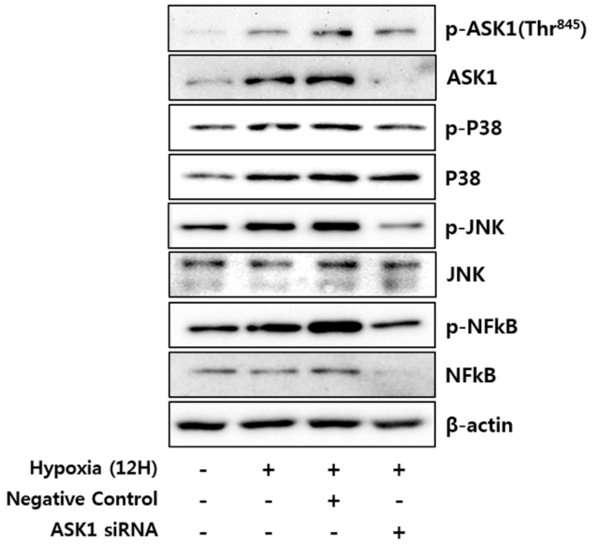

Figure S5. ASK1-downstream molecules were measured by immunoblot followed by ASK1 siRNA transfection under hypoxic conditions.

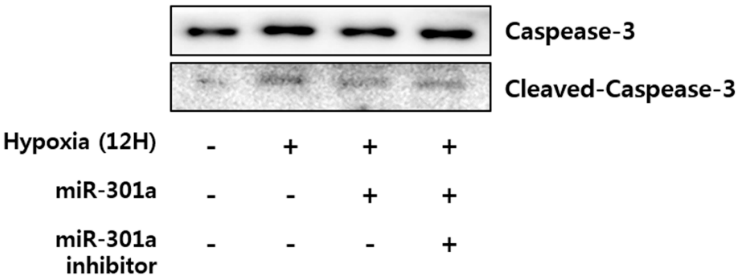

Figure S6. The levels of caspase-3 were detected by immunoblot.
